# Identification and Expression Analysis of the Alfin-like Gene Family in Tomato and the Role of *SlAL3* in Salt and Drought Stresses

**DOI:** 10.3390/plants12152829

**Published:** 2023-07-31

**Authors:** Ruixin Jin, Juan Wang, Bin Guo, Tao Yang, Jiahui Hu, Baike Wang, Qinghui Yu

**Affiliations:** 1 College of Life Science and Technology, Xinjiang University, Urumqi 830046, China; 18099304389@163.com (R.J.); drjuanwang@126.com (J.W.); 2 Institute of Horticulture Crops, Xinjiang Academy of Agricultural Sciences (Key Laboratory of Genome Research and Genetic Improvement of Xinjiang Characteristic Fruits and Vegetables), Urumqi 830091, China; yangtao_xj@sina.com (T.Y.); hujiahui1110@163.com (J.H.); 3 The State Key Laboratory of Genetic Improvement and Germplasm Innovation of Crop Resistance in Arid Desert Regions (Preparation), Urumqi 830091, China; gb@xjau.edu.cn; 4 College of Computer and Information Engineering, Xinjiang Agricultural University, Urumqi 830052, China

**Keywords:** AL transcription factor, tomato, evolutionary analyses, expression patterns, abiotic stresses, *SlAL3*

## Abstract

Alfin-like (AL) transcription factors are a family of plant-specific genes with a PHD-finger-like structural domain at the C-terminus and a DUF3594 structural domain at the N-terminus that play important roles in plant development and stress response. In the present study, genome-wide identification and analysis were performed of the AL protein family in cultivated tomato (*Solanum lycopersicum*) and three wild relatives (*S. pennellii*, *S. pimpinellifolium,* and *S. lycopersicoides*) to evaluate their response to different abiotic stresses. A total of 39 *ALs* were identified and classified into four groups and based on phylogenetic tree and evolutionary analysis were shown to have formed prior to the differentiation of monocotyledons and dicots. Moreover, *cis*-acting element analysis revealed that various phytohormone response and abiotic stress response elements were highly existed in tomato. In addition, further analysis of the *SlAL3* gene revealed that its expression was induced by drought and salt stresses and localized to the nucleus. In conclusion, our findings concerning *AL* genes provide useful information for further studies on their functions and regulatory mechanisms and provide theoretical references for studying *AL* gene response to abiotic stresses in plants.

## 1. Introduction

As sessile organisms, plants constantly suffer multiple abiotic stresses at different stages of growth, resulting in massive annual losses in crop production [1,2]. Therefore, a variety of ways to mobilize a series of stress-responsive genes has evolved to achieve broad adaptations over a long evolutionary period to allow plants to survive inevitable environmental stresses and maintain normal growth and development. To combat environmental stimuli or stressors, sophisticated genetic mechanisms regulating gene expression are mediated by accurate transcriptional control [3]. Transcription factors (TFs) regulate the transcriptional control of gene expression by binding directly to the corresponding *cis*-acting element, and these regulatory factors play pivotal roles in several signaling networks [4]. Among the TFs, the stress-responsive TFs are dedicated to increasing plant adaptation to adverse environments via response stressors such as cold, salt, and drought as well as defense responses against invading pathogens [5,6].

Alfin-like (AL) TFs are a group of proteins presumed to play crucial roles in multiple physiological processes related to plant development and stress responses such as seed germination, root development, root hair elongation, and meristem development [7,8,9]. AL proteins are characterized by two conserved domains, a DUF3594 domain and a PHD-finger structural domain, located at their N-termini and C-termini, respectively. Although the DUF3594 domain is functionally uncharacterized, the highly conserved nature of the DUF3594 domain in different species indicates that AL proteins probably have fundamental biological functions in plants [10]. Previous reports revealed the characteristics of the PHD-finger, which is involved in efficiently binding to the conserved consensus binding sequence GNGGTG or GTGGNG in the promoter of the target gene, marking the transcription start sites of virtually all active genes and recognizing H3 tails trimethylated on lysine 4 (H3K4me3) [11,12].

To date, a limited number of studies have identified and characterized proteins of the AL family in plants. Initially, the first *AL* gene discovered 30 years ago was found to be involved in numerous metabolic and physiological processes related to plant development and stress responses in *alfalfa* [13]. Another relevant study revealed that *Alfin1* contained a specific DNA-binding sequence DNA [11]. Since then, comprehensive molecular evolutionary analyses have been conducted in two *Arabidopsis* species (*A. lyrata* and *A. thaliana*) and a close salt-tolerant relative, *Thellungiella halophila*, indicating that the predicted proteins of the AL family undergo adaptive evolution in members of the *AL* gene family [10]. Furthermore, the *AtAL6* gene was reported to confer root hair elongation under phosphate deficiency conditions, exhibiting a novel physiological function [7]. Subsequently, to elucidate the exact functions of the 15 identified proteins of the *AL* gene family for *Brassica rapa*, further expression profile analysis was conducted under biotic and abiotic stresses, and the results were compared, suggesting that 10 *BrALs* TFs showed responses to detrimental environments and biotic invasion [14]. Likewise, the results of genome-wide investigations of *B. oleracea* revealed organ-specific expression of the corresponding 12 *BoALs* genes, and 10 *BoALs* were shown to induce at least one abiotic stress and increase the endurance of *B. oleracea* to abiotic stresses, such as cold, salinity, drought, and abscisic acid treatment [15]. More recently, in maize, 18 *ZmALs* genes classified into four groups by systematic genome-wide identification were predicted, and expression analysis, focused on their roles under salt, cold and drought stress conditions in particular, was fully performed, suggesting that the AL family could be considered a reservoir of stress-responsive genes with great potential for the genetic improvement of stress tolerance in maize [16]. With the rapid generation of plant whole genome sequences and the development of bioinformatics technology, comprehensive analyses of the evolution and functional diversification of *AL* gene families are increasingly supplying information emphasizing the important roles of *ALs* in plants during abiotic stresses.

Tomato, belonging to the nightshade family (*Solanaceae*), is a staple vegetable crop around the world and has been widely used in genetic and other types of basic biological research due to its diploid mode of inheritance, ease of seed and clonal propagation, efficient sexual hybridization, and short generation period [17]. With the completion and supplementation of the International Tomato Genome Sequencing Project, the genomic information available for four tomato species including *S. lycopersicum*, *S. pennellii*, *S. pimpinellifolium,* and *S. lycopersicoides*, provides opportunities for identifying and classifying genes in these tomatoes and presents a basis for systematic expression profiling and in-depth biochemical, physiological, and functional studies, in addition to studies of the evolution of the AL family [18,19,20]. Despite the contributions of ALs to stress tolerance in plants, no systematic genome-wide identification and expression analyses of the *AL* gene family in tomato have been carried out so far. In the present study, we systematically characterized *AL* genes in tomato, based on the most recent genome sequences of one cultivated tomato (*S. lycopersicum*) and three wild relatives (*S. pennellii*, *S. pimpinellifolium,* and *S. lycopersicoides*). The results of a detailed analysis examining protein features, gene structures, phylogenetics, chromosomal localization, gene duplication, *cis*-acting elements, selective pressures, and expression profiles using bioinformatic methods offered insight into their potential functions in response to various challenging survival environments. In brief, our results concerning *AL* genes supply useful information for further functional studies and evaluations of regulatory mechanisms and will serve as a theoretical reference for research on *AL* genes on plant abiotic resistance.

## 2. Results 

### 2.1. Identification and Characteristics of ALs in Tomato

To identify tomato *AL* gene candidates, we applied a hidden Markov model (HMM)-based approach and the protein sequence profile of the DUF3594 domain (PF12165) and the PHD zinc-finger-like motif (PF00628). The existence of these domains in the putative sequences was verified by hmmscan (https://www.ebi.ac.uk/Tools/hmmer/search/hmmscan, accessed on 16 July 2022). In general, 11, 9, 11, and 8 *AL* genes (*ALs*) were identified in *S. lycopersicum*, *S. pimpinellifolium*, *S. pennellii,* and *S. lycopersicoides*, respectively. To facilitate further analysis, these genes were annotated according to their physical location as *SlAL1* to *SlAL11*, *SpiAL1* to *SpiAL9*, *SpAL1* to *SpAL11,* and *SlydAL1* to *SlydAL8*. The physicochemical properties of the *AL* genes were quantitatively evaluated based on the online analysis software ExPASY (https://www.expasy.org/, accessed on 25 July 2023), including the calculation of protein length (PL), molecular weight (MW), isoelectric point (PI), the instability index, and the mean value of hydrophilicity (GRAVY). As shown in Supplementary Table S1, the proteins encoded by the 39 *AL* genes exhibited similar physicochemical properties. The majority of the AL proteins in these four species encode peptides of 213 (*SpiAL1*) to 298 (*SlydAL2*) amino acids, with a predicted molecular weight ranging from 24.0 kDa to 33.9 kDa. Physicochemical property analysis revealed that all AL proteins in tomato have relatively low isoelectric points (pI < 7), between 4.73 and 5.85, and show an acidic nature. Regarding the grand average of hydropathicity, the results indicated that these deduced proteins are hydrophilic proteins, all showing indices < 0. To evaluate protein instability, instability index (II) scores were obtained, ranging from 38.56 to 63.40, with only one protein (*SlydAL6*) presenting a value below 40. In addition, subcellular localization analysis indicated that most AL proteins localize to the nucleus, with the exception of *SlAL2*, *SpiAL2*, *SpiAL8*, *SpAL2,* and *SlydAL3*, which localize to the cytoplasm or extracellular space. The above results are predictions, and the genes other than *SlAL3* were not analyzed and validated.

### 2.2. Phylogenetic Analyses, Gene Structure, and Conserved Motif Distributions of ALs in Tomato

**Figure 1:**
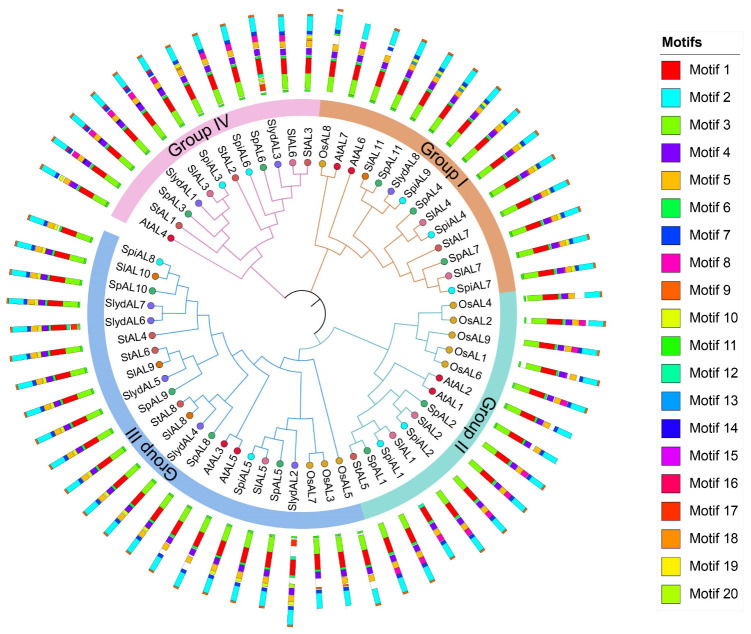
Phylogenetic analysis of AL proteins from *Arabidopsis thaliana* (At), *Oryza sativa* (Os), *Solanum tuberosum* (St), and tomatoes. The rooted maximum likelihood tree was constructed from alignments of 63 AL protein sequences from *A. thaliana* (7), *O. sativa* (9), *S. tuberosum* (8), and tomatoes (39) under the LG + G + F model with 1000 bootstrap replications. The outermost circle shows 20 motif models of *AL* genes in different species.

To better understand and examine the evolutionary relationships among AL family members, a phylogenetic tree was built based on the similarity of their protein sequences via the neighbor-joining method. According to the phylogenetic tree, the AL family was categorized into four main clades (Figure 1 and Figure S1), and the *AL* clustering pattern and clades designated Groups I–IV exhibited high similarities to those in previous reports on *Arabidopsis* [10]. Each group contained tomato *AL* members, and the number of *AL* members in different subfamilies varied greatly. Interestingly, there were 10 members from four tomato species in Group I, which was consistent with *AtAL6* from *Arabidopsis* shown to be involved in root hair elongation during phosphate deficiency [7]. Moreover, among the four groups, Group III had the largest number of *AL* members (15) in tomato and presented high sequence similarity to *AtAL5*, which confers remarkable cold, drought, and salt tolerance in transgenic plants [21]. In addition, the observed evolutionary relationships indicated that the AL proteins of tomato showed a close affinity with AtAL proteins but had a distant relationship with the ZmALs and OsALs within the same group. These relationships may be partly explained by the fact that tomato and *Arabidopsis* are dicotyledons, while rice is monocotyledons.

### 2.3. Gene Structure and Conserved Motif Analysis of Tomato ALs

**Figure 2:**
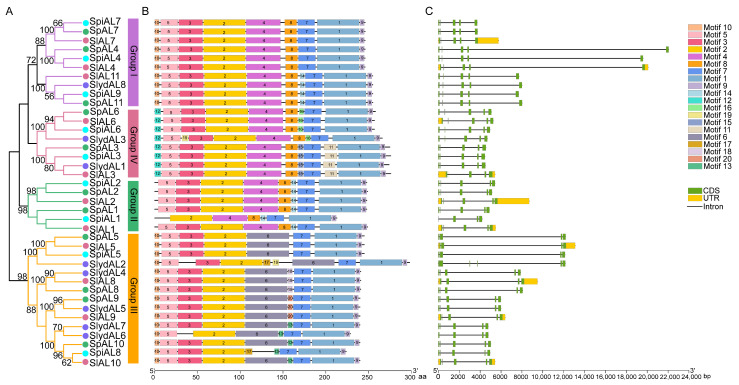
Motif and gene structure analysis of tomato ALs. (**A**) Maximum likelihood (ML) tree generated by MEGA-X under the LG + G model with bootstrapping analysis (1000 replicates). (**B**) Conserved domain and motifs of tomato ALs were conducted with Hmmsearch. Different color boxes represent different motifs (20). (**C**) Exon–intron structures of tomato *ALs* generated by GSDS online software (http://gsds.gao-lab.org/, accessed on 25 July 2023). The CDS region is represented by a green box, the UTR region is represented by a yellow box and introns are represented by a black line.

To gain insight into the evolutionary relationships between AL proteins from the four species of tomato, another phylogenetic tree was constructed, and further conserved motif and intron–exon analyses were performed to reveal more information about *AL* gene similarities as shown in Figure 2 (Supplementary Figure S2). These analyses showed that they clustered into four groups in the tree and that the most closely related members of the family tree showed similar exon–intron arrangements and shared the same motifs within the same taxa (Figure 2A). Further motif analysis showed that the 39 AL proteins exhibited high similarity, such as motif 1, motif 2, motif 7, and motif 9, which were present in all AL proteins and may constitute the AL domain (Figure 2B). However, the members of each AL subgroup contained multiple specific domains; for example, motif 6 only existed in Group III. The diversity of gene structure indicates a need for the evolution of multigene families [22], so the analysis of gene structure may provide pivotal information about gene function, organization, and evolution. To obtain insight into the structural diversity of the genes, their structural organization was analyzed based on comparing the full-length cDNAs of the *AL* genes. The divergence of exon and intron patterns showed that there were three to four introns among the 39 *ALs* of tomato, and the *AL* genes with similar exon and intron architectures were in the same group, indicating that *AL* gene structure is strongly correlated with the observed phylogenetic relationship (Figure 2C).

### 2.4. Cis-Acting Element Analysis of Tomato ALs

**Figure 3:**
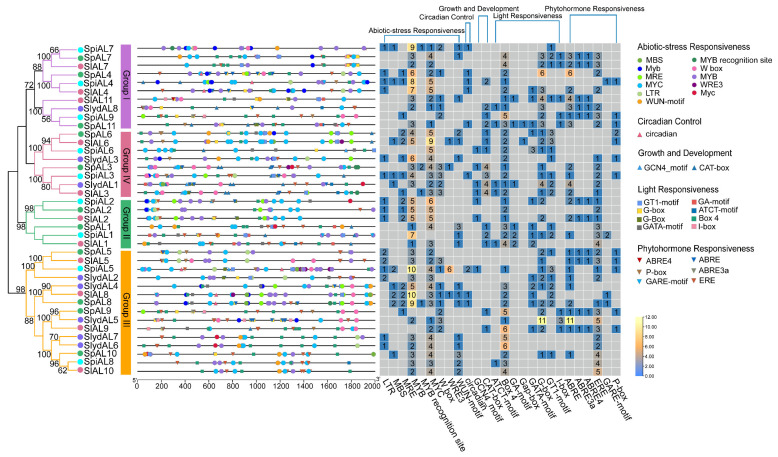
*Cis*-acting elements in *AL* genes. *Cis*-acting elements were predicted with the PlantCARE using the 2000 bp upstream region of the start codon of each gene extracted from the corresponding genome sequence. Numbers in boxes are the numbers of each element in the promoter region.

To further elucidate the possible regulatory mechanisms underlying the tomato *AL* gene responses to abiotic stress, *cis*-acting elements were investigated in promoter regions, defined as the regions 2000 bp upstream of a transcription start site (TSS). The results demonstrated that the upstream sequences of *AL* genes contained various potential *cis*-acting elements, including MYB, MBS, MYC, and LTR elements, which conferred responses to abiotic stresses at the transcriptional level. These findings showed that *ALs* may be active and play a crucial role under abiotic stress. In addition to abiotic stress elements, the remaining *cis*-acting elements were characterized and grouped into four categories correlated with the regulation of phytohormone responsiveness (20.49%, 175/854), light responsiveness (27.40%, 234/854), development (4.45%, 38/854), and circadian control (1.17%, 10/854) (Figure 3 and Supplementary Table S2). Among the hormone-related elements, ERE elements were present in the promoters of 31 *AL* genes, and ABRE-related elements existed in 25, 11, and 11 *ALs* in tomato, respectively. The major light responsive elements contained box 4 and G-boxes, which were found in 35 and 28 *ALs* in tomato respectively. Additionally, the last category contained plant growth-related elements such as GCN4 motifs and CAT boxes (Supplementary Table S3). These results implied that *AL* genes may play significant roles in the regulation of responses to various abiotic stresses and plant growth. The above results present the function of the *cis*-acting elements, although not all of them were confirmed, but they also provide potential applications for the development of plants with corresponding resistance.

### 2.5. Chromosomal Location and Gene Duplication of AL Genes in Tomatoes 

**Figure 4:**
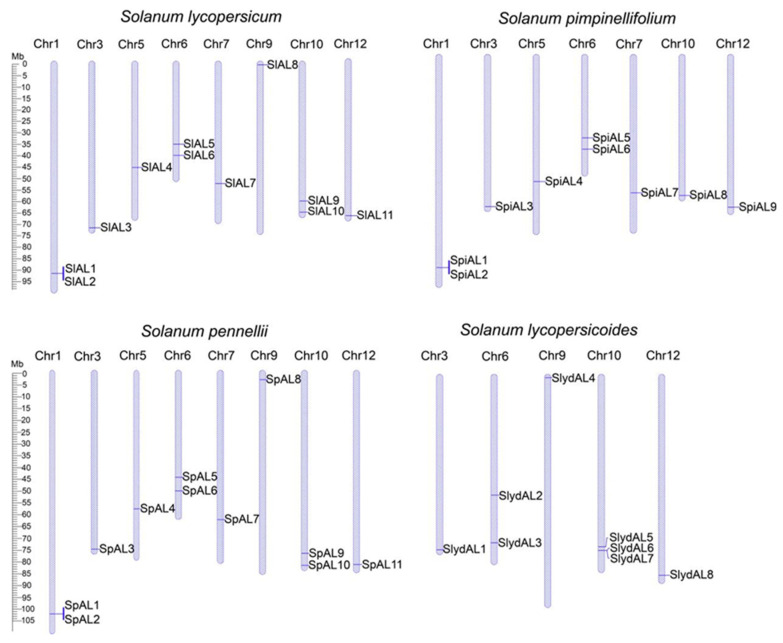
Chromosomal locations of 39 *ALs* in four tomato species. The chromosomes are represented by blue columns and the chromosome number is displayed at the top of each chromosome. The size of the chromosome is listed in metabases (Mb).

The genomic distribution of *ALs* was determined by examining the chromosomal distribution of the AL family in four tomato varieties (Figure 4). The results showed that the 39 *AL* genes were distributed on eight chromosomes, with the exception of chromosomes 2, 4, 8, and 11. Among the varieties, the distribution of *ALs* in *S. lycopersicum* and *S. pennellii* was very similar, with two *AL* genes distributed on each of chromosomes 1, 6, and 10 and one *AL* gene on each of chromosomes 3, 5, 7, 9, and 12; in *S. pimpinellifolium*, two *AL* genes were distributed on each of chromosomes 1 and 6, and chromosomes 3, 5, 7, 10, and 12 each contained one *AL* gene; in *S. lycopersicoides*, chromosomes 3, 9, and 12 each presented one *AL* gene, chromosome 6 had two *AL* genes, and chromosome 10 presented three *AL* genes. Almost all of the *AL* genes were distributed at both ends of the chromosomes.

**Figure 5:**
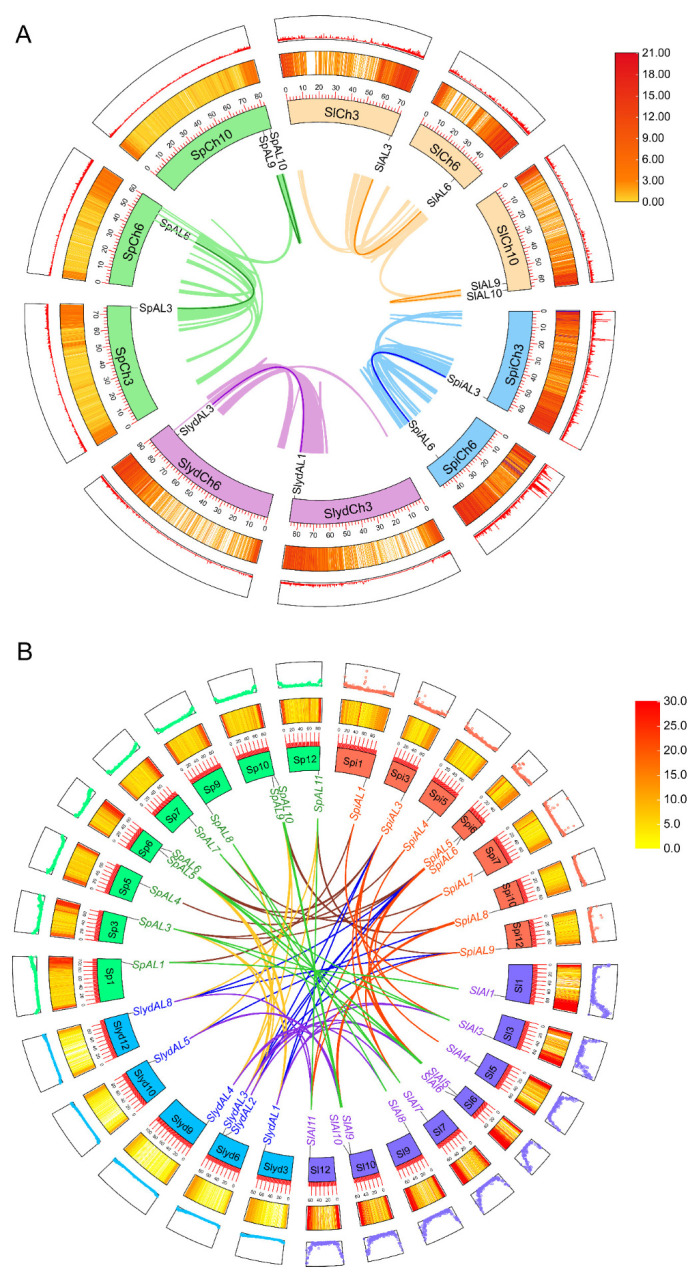
Intraspecies synteny analysis of AL gene in tomatoes. (**A**) Intraspecific collinearity analysis of four tomato species. (**B**) Analysis of collinearity among four tomato species. The circles are from inside to outside: the innermost circle is the location of the chromosome where the gene is located, and the number on the chromosome represents the length of that chromosome; both the middle circle and the outermost circle indicate the gene density on that chromosome, with the middle circle shown as a heat map and the outermost circle shown as a line (**A**) and a circle (**B**). Both indicate the magnitude of gene density, the ends of the lines represent directly homologous AL genes, and the different colored lines represent different evolutionary patterns.

The expansion of gene families can be attributed to gene duplication, including both fragmentary and tandem duplication of genes [23]. Based on the definition of replication, among the four tomato varieties, only three genes were located at extremely similar positions at the chromosome level (*SlAL1*/*SlAL2*, *SpiAL1*/*SpiAL2* and *SpAL1*/*SpAL2*) and were presumed to have undergone tandem replication events, while the six pairs of genes distributed on different chromosomes implied that they had undergone fragmental replication (Figure 5A and Supplementary Table S4). We also calculated synonymous substitution rates (Ks), assuming a rate of 1.5 × 10^−8^/synonymous locus/year in dicotyledons [24], to assess the timing of *AL* gene diversification within the genome. *S. pennellii* is thought to have originated 21.67 Mya to 44 Mya. In *S. lycopersicum SlAL3*/*SlAL6*, the duplication of the *SlAL3*/*SlAL6* homologous gene pair in *S. lycopersicum* occurred approximately 22.67 Mya, and the duplication of the *SlAL9* and *SlAL10* homologous gene pairs occurred approximately 38 Mya. The duplication of the *SpiAL3* and *SpiAL6* homologous gene pairs in *S. pimpinellifolium* occurred 22.33 Mya. However, the tandem duplication of the three homologous gene pairs is thought to have occurred 9.00 to 10.00 Mya. To further determine the relationship between replication events and selection pressure, we calculated the Ka/Ks ratios of the identified biparental gene pairs. As shown in Supplementary Table S4, the selection pressure analysis showed that the Ka/Ks ratios of these AL replication events were all less than 1, implying that the tomato *AL* genes were evolutionarily influenced by purifying selection. A similar result was found in soybean [25]. Combined with these results, it appears that tandem replication primarily drives the amplification of *AL* genes in the tomato genome, implying that *AL* genes occur slowly after genome-wide replication in *Solanaceae*.

### 2.6. Collinearity and Evolutionary Analysis of AL Genes in Tomato

To further discover the origin of the AL family, interspecific collinearity analysis was carried out among the genomes of the four species of tomato, among which the identified gene pairs originated via segmental duplication, and it was notable that more *AL* orthologous genes were distributed at both ends of chromosomes, with higher gene density near the ends of chromosomes, and lower gene density in centromeric regions (Figure 5B). Sixty-nine paralogues were found to exist among the species, including 15 in *S. lycopersicum* and *S. pennellii* and 12 in *S. lycopersicum* and *S. lycopersicoides*. In addition, a total of 11 orthologous gene pairs were identified between *S. lycopersicoides* and *S. pennellii*, 12 in *S. pimpinellifolium* and *S. lycopersicum*, 10 within *S. pimpinellifolium* and *S. pennellii*, while the lowest ortholog number (9) was found between *S. pimpinellifolium* and *S. lycopersicoides* (Figure 5B and Supplementary Table S5).

**Figure 6:**
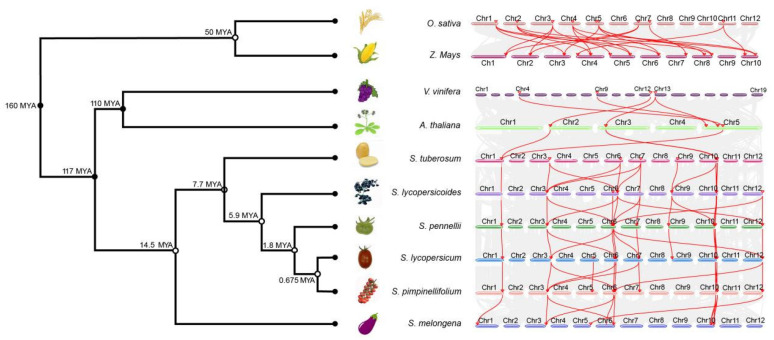
Synteny analysis of *AL* genes between four tomato species and six other plant species. The gray lines in the background represent the collinear blocks within tomato and other plant genomes, while the red lines highlight the syntenic *AL* gene pairs.

To determine the evolutionary origins and orthologous relationships of the *AL* gene family, interspecies collinearity analysis was carried out among the four tomato species and six representative species, including four dicots (*A. thaliana*, *S. tuberosum*, *S. melongena,* and *Vitis vinifera*) and two monocots (*O. sativa* and *Z. mays*). The divergence time results and collinearity analysis indicated similar homology between species in *Solanaceae*, *A. thaliana,* and *V. vinifera* compared to that between rice and maize (Figure 6). The present study revealed that the AL family expanded with the whole genome replication of angiosperms. Compared to dicots, the number of homologous members of the AL family was greater than that in monocots and the evolutionary speed was accelerated. Grapevine, an ancient ancestor that has remained highly conserved during the evolutionary process [26], has four *AL* genes and is homologous with several *AL* genes in *Solanaceae*. The results indicated that the AL family in dicotyledons may have arisen via the duplication of a single *AL* gene in the ancestral species.

### 2.7. Expression Patterns of AL Genes in Tomato

**Figure 7:**
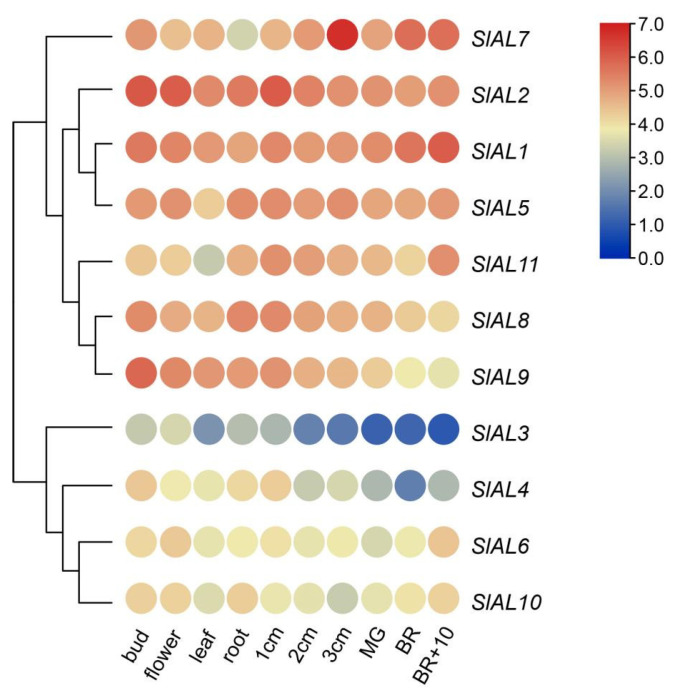
Heat map of tissue-specific expression of *AL* genes in tomato. Fully opened flowers, leaf, roots, 1 cm fruits, 2 cm fruits, 3 cm fruits, unopened flower buds, mature green fruits (MG), breaker fruits (BR), and breaker + 10 fruits (BR + 10).

To further explore the tissue specificity of *AL* genes in tomato, available transcriptome data were used for the present study (TomExpress, http://tomexpress.toulouse.inra.fr/, accessed on 25 July 2022). The *AL* gene expression patterns observed in different tissues showed that all *SlALs* presented obvious tissue-specific expression, and they were divided into two groups according to their expression (Figure 7, Supplementary Table S6). The paralogous gene pair *SlAL1* and *SlAL2* presented relatively high expression levels in all tissues, especially in buds and flowers, which indicated that these two *AL* genes may participate in flower development. Additionally, gene pairs reflecting one tandem duplication and some duplicated pairs exhibited different expression patterns in organs. For instance, *SlAL3* showed a rise–fall tendency, with the relative content beginning to decrease after the fruit reached a diameter of 1 cm, whereas the *SlAL6* a duplicate gene of *SlAL3*, showed a higher expression level during fruit ripening, indicating subfunctionalization after a duplication event.

**Figure 8:**
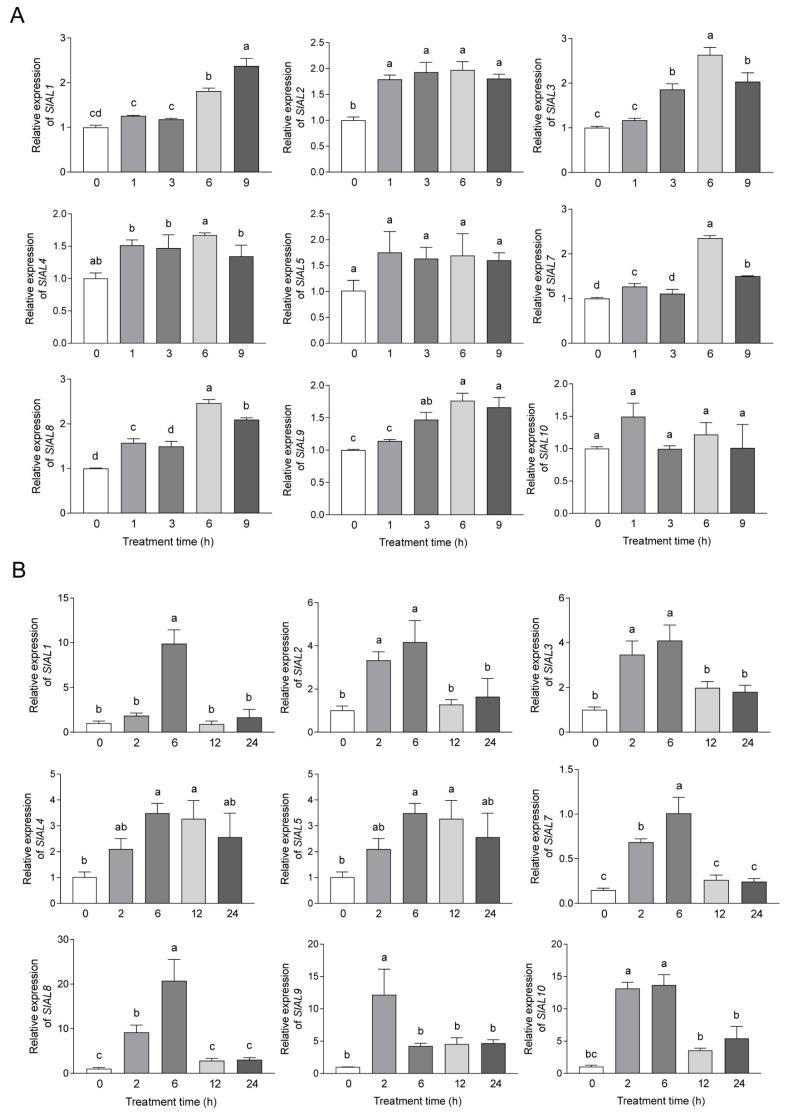
qRT-PCR validation of the *AL* genes from *S. lycopersicum* under simulation of drought and salt stress. (**A**) The horizontal coordinates of the graph are the different periods of 300 mmol/L mannitol treatment, and the vertical coordinates are the relative expression of each *AL* gene; (**B**) The horizontal coordinates of the graph are the different periods of 200 mmol/L NaCl treatment, and the vertical coordinates are the relative expression of each *AL* gene. The standard deviations of three independent biological replicates are indicated by error lines. Different lower-case letters indicate significant differences between means as measured by ANOVA followed by Duncan’s multiple range test (*p* < 0.05).

To further determine whether abiotic stress affects the expression levels of *AL* genes, nine *SlAL* candidate genes were selected, and the response characteristics of tomato genes to simulated drought stress were explored by qRT-PCR. The relative expression of *SlALs* in osmotically stressed leaves at the 0, 1, 3, 6, and 9 h time points was verified, with 0 h treatment as the control group. The results showed that the relative expression of all nine *AL* genes was differentially upregulated with increasing drought treatment time compared with the control, suggesting that these nine *AL* genes may play a specific role under osmotic stress conditions (Figure 8A). Except for *SlAL1*, *SlAL5*, and *SlAL10*, all other *AL* genes showed the highest relative expression at the treatment time of 6 h. *SlAL1* showed a generally upward trend from 0 h to 9 h, and *SlAL5* and *SlAL10* showed no significant difference compared with the control group. Subsequently, we explored the response characteristics of the nine *SlAL* candidate genes under salt stress by qRT-PCR. The relative expression patterns of *SlALs* in salt stressed leaves at 0, 2, 6, 12, and 24 h were verified, with 0 h treatment as the control group. The results showed that the nine *AL* genes were differentially regulated with an increasing salt treatment time, indicating that the nine *AL* genes may also play specific roles under salt stress conditions (Figure 8B). The relative expression levels of the *SlAL1*, *SlAL2*, *SlAL3*, *SlAL4*, *SlAL5*, *SlAL7*, and *SlAL8* genes gradually increased compared with the control until reaching a maximum at 6 h, after which their relative expression levels decreased. The relative expression level of *SlAL9* peaked at 2 h and then decreased. The relative expression of *SlAL10* was more than tenfold higher than that of the control at both 2 and 6 h of treatment, and the relative expression levels at the subsequent time points of 12 and 24 h differed less from that of the control. Overall, most of the *SlAL* genes were responsive to external osmotic and salt stress and showed a positive expression pattern in response to osmotic stress versus salt stress.

### 2.8. Subcellular Localization Analysis

**Figure 9:**
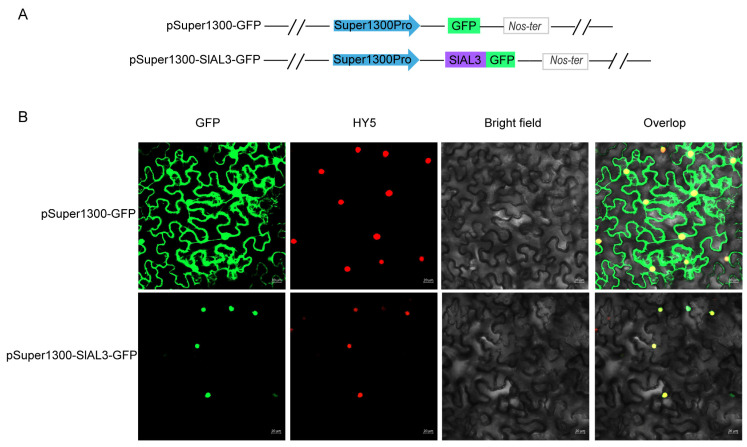
Subcellular localization analysis of SlAL3. (**A**) Schematic diagrams of pSuper1300-SlAL3-GFP and pSuper1300-GFP (positive control) structures; (**B**) Images show the GFP signals of both structures in transgenic tobacco. Green fluorescence was observed under confocal microscopy. HY5: nuclear localization protein; GFP: green fluorescent protein; bright field: visible light; overlap: merged bright field with HY5 and GFP. bar = 20 μm.

The subcellular localization of SlAL3 was predicted by the online software WoLF PSORT (https://www.genscript.com/wolf-psort.html, accessed on 16 July 2022), which showed that SlAL3 was associated with the nucleus. Further verification by the transient expression of pSuper1300-SlAL3-GFP in *Nicotiana benthamiana* showed that strong green fluorescence was exclusively localized to the nucleus, which may support a role of SlAL3 as a nuclear transcription factor (Figure 9).

## 3. Discussion

Alfin-like TFs play important roles in regulating signals related to plant salt tolerance [8,11,27]. There are two domains, a PHD-finger domain at C-terminus and a DUF3594 domain located at the N-terminus [8,27,28]. There is a complex regulatory network of AL transcription factors associated with abiotic stress tolerance. The first report related to these genes was published 30 years ago and showed that *AL* genes were involved in *alfalfa* plant development and stress responses [13]. Subsequently, AL proteins were found to be involved in abiotic stresses in many plants, including roles such as enhancing *MsPRP2* gene expression in *alfalfa* roots, thus improving plant salt tolerance [11]. In *Arabidopsis, AL1* and *AL5* bind to the promoter regions of target genes and repress corresponding negative regulators, thus improving plant tolerance to abiotic stresses [21,29]. *Arabidopsis AL6* promotes the regulation of root hair elongation in the presence of phosphorus deficiency [7,10]. Based on a combined molecular biology and genetic informatics analysis, the first *AL* gene family and the characteristics and functions of this family were identified and studied in detail in the model plant *Arabidopsis* [7]. The *AL* gene family has been reported in many crops; in contrast, the genome-wide identification and annotation of *AL* gene have not been previously reported in tomato. Our results contribute to a better understanding of the complexity of the *AL* gene family and will facilitate functional characterization in future studies.

In the present study, we accurately identified a total of 39 *AL* genes based on the existence of HMM profiles of *AL* genes from high-quality tomato genome sequences including sequences from three wild species, according to information on physical locations in the genome. The identified numbers of *AL* genes in tomato (11 in *S. lycopersicum* and *S. pennellii*, 8 in *S. lycopersicoides*, 9 in *S. pimpinellifolium*) were roughly similar to those in *Arabidopsis* [30], rice [31], and *B. oleracea* [15], which was less than that in *B. rapa* [14] and maize [16]. Several tomato *AL* genes homologous to genes of *A. thaliana*, *B. oleracea*, *B. rapa*, and *Z. mays* have also been found to possess similar basic characteristics, indicating that this gene family was formed before the differentiation of monocotyledonous and dicotyledonous plants.

To evaluate the evolutionary relationships of the *AL* gene family across different species, both dicotyledonous (5) and monocotyledonous (2) plants were selected for further comparisons. The results showed that the *AL* genes of tomato were more homogenous than those of other dicotyledonous species and less homogenous than those of two monocotyledonous species, which further verified the results showing more collinear tomato *AL* gene pairs among dicotyledonous plants, rather than monocotyledonous plants. Following interspecific phylogenetic tree construction in tomato, the grouping and evolutionary relationships of the tomato *AL* gene family were determined. Thirty-nine AL proteins were divided into four subgroups in this study, with most members in a particular subgroup showing the same intron pattern and conserved motifs, suggesting the regulation of similar biofunctions. The phylogenetic topology diagram revealed 20 highly conserved amino acid motifs in the 39 AL proteins. The results of protein motif analysis and the composition of each AL protein family were similar to those found in previous studies performed on *B. oleracea* and *B. rapa* [14,15]. Depending on the continuous updating and improvement of databases and annotation information, genome-wide identification analyses are continuously improving, and the data analysis will be different. Signature motifs 1, 2, 7, and 9 were found in almost all AL proteins and were always adjacent to each other, constituting the AL domain. An increasing number of studies are revealing that introns exert gene function by affecting the regulation of gene expression [32] and intragenic recombination [33]. Reduced intron numbers in stress responsive genes were recorded, as found in the trehalose-6-phosphate synthase gene family which plays an important role in abiotic stress and metabolic regulation [34]. The conserved similar exon/intron organization of AL members in the same clade including three to four introns, as similarly reported in the relevant literature [15], together with the phylogenetic analysis results, could support the reliability of the clade classifications and imply similar functions in stress tolerance. From a gene structure standpoint, these findings support the high expression and anti-stress functions of *AL* genes.

Promoter analysis, an important TF analysis method, confirmed that multiple *cis*-acting elements play a functional role in regulating gene expression under stress [35,36]. *Cis*-acting elements are important regulatory components that can be bound by the transcription factors to regulate their expression. *Cis*-acting regulatory element analysis showed that various elements participating in phytohormone responsiveness, plant growth and development, light responsiveness, and abiotic stress responsiveness, such as MYB, LTR, MBS, and MYC elements, were more abundant in tomato. These results were consistent with the high relative expression levels of *SlAL1*, *SlAL2*, *SlAL3,* and *SlAL8*, with three MYC elements, observed under drought stress. MYC elements are present in the promoters of all *SlAL* genes, while other elements are present in only some genes. Surprisingly, *SlAL4*, with five MYC elements, showed no significant difference in expression under osmotic stress, and we speculate that the MYC binding sites in *SlAL4* do not perform biological functions. Due to the abundant stress-related *cis*-acting elements in the promoter regions of the other genes, these genes showed pervasive expression after drought treatment. Additionally, a previous study revealed that the expression of *BoAL1*, *5*, *7*, *9*, and *10* was higher under drought stress at the sixth time point during drought stress treatment [14], and the expression level of all drought condition induced *BoAL* genes peaked at the sixth time point of drought stress [15], which was in keeping with our findings. The *SlAL* genes were induced by drought stress, and their expression levels were highest at 6 h under drought stress and showed different patterns in most cases.

The expression patterns of *AL* genes in different tissues have been described in many species, including transgenic *alfalfa* [37] and *A. thaliana* [10]. In particular, roots have the ability to detect changes in the osmotic potential of the soil, allowing them to respond appropriately to drought and high salinity, as claimed in a previous study [38]. For example, the overexpression of *Alfifin1* contributed to root growth in transgenic *alfalfa*, and *AtAL6* TFs control root hair elongation in phosphate-deficient conditions, while *AtAL7* overexpression and T-DNA insertion mutants played a negative role in salt tolerance during early seedling development. Roots have the ability to detect changes in the osmotic potential of the soil, allowing them to respond appropriately to drought and high salinity [38]. Here, *AL* gene expression exhibited three obvious characteristics: (i) the expression of most genes (8/11) is higher in roots than in leaves; (ii) in the fruits, the expression of genes such as *SlAL2*, *SlAL3*, *SlAL8*, and *SlAL9* gradually decreases from the beginning of fruit growth until the breaker + 10 fruit stage; (iii) there is no difference in the expression of genes such as *SlAL5* and *SlAL10* between different organs and stages. The *AL* genes showing similar expression patterns, may hint at similarities in structures, redundancies in functions, and shared induction mechanisms.

Through comparative genome analysis, we gained deeper insight into the paralogous and orthologous relationships among the members of the *AL* gene family, confirming gene expansion resulting from gene duplications and revealing their intraspecific and interspecific collinearity. Segmental duplication, tandem duplication, and transposition events were the main reasons for gene family expansion [39]. Based on the analysis of replication events, it can be concluded that *ALs* in tomato underwent two genome-wide replications, resulting in six segmental and three tandem replications. The number of *AL* genes with tandem duplications accounted for 15.38% (6/39) of all *AL* genes, while the number of *AL* genes with segmental duplications accounted for 30.77% (12/39) of the total. This indicates that segmental duplication is the main mode of expansion of tomato *ALs* in the duplication events of tomato *AL* genes. Intriguingly, while four paralogous gene pairs acquired analogous intron/exon structures and motif components during evolution, their function seems to have diversified throughout evolution.

In addition, the *AL* gene distribution followed a high-level pattern of microsynteny, with some genes presenting close physical locations on a single chromosome, indicating that their corresponding syntenic blocks in other species, such as in *S. pimpinellifolium* (*SpiAL5* and *SpiAL6*) and in *S. pennellii* (*SpAL5* and *SpAL6*), would also be located close to each other on the same chromosome, and they were also contiguous in the related genomes. The syntenic blocks of segmentally duplicated *AL* genes are likely to be conserved as well. For instance, the *AL3* and *AL6* genes are segmentally duplicated genes in *S. pimpinellifolium*; and their corresponding syntenic blocks in other species are identical, as observed for *SpAL3* and *SpAL6* in *S. pennellii*, and *SlAL3* and *SlAL6* in *S. lycopersicum*. Additionally, segmentally duplicated *AL* gene syntenic blocks are likely to be conserved. For example, the *AL3* and *AL6* genes of *S. pimpinellifolium* are segmentally duplicated genes; and their corresponding syntenic blocks in other species, such as *SpAL3* and *SpAL6* in *S. pennellii* and *SlAL3* and *SlAL6* in *S. lycopersicum*, are identical. Interestingly, these *AL* genes had relatively similar intron–exon patterns and the estimated divergence time of the species to which the gene belongs was recent, usually distributed in the same phylogenetic group or phylogenetic sister group.

## 4. Materials and Methods

### 4.1. Retrieval and Identification of Putative AL Proteins in Four Tomato Species 

All AL protein sequences were downloaded from the Tomato Genome sequencing projects databases (https://solgenomics.net/, accessed on 7 July 2022). The hidden Markov model (HMM) profile for the DUF3594 domain (PF12165) and the PHD zinc-finger-like motif (PF00628) was produced from the Pfam protein family database (http://Pfam.xfam.org, accessed on 7 July 2022). To identify the potential *AL* genes in tomatoes, all existing *AL* genes in *A. thaliana* were obtained from the *Arabidopsis* Information Resource (TAIR, https://www.arabidopsis.org/index.jsp, accessed on 8 July 2022) for further analysis. Viewing the *AtAL* gene as a target sequence, tomato *AL* genes with two conserved domains were obtained for further study, and genes without conserved domains were removed. Then, BLAST at SMART (http://smart.embl.de/, accessed on 8 July 2022) was used to determine whether they belonged to the *AL* gene family. The physicochemical characteristics of the AL proteins were analyzed with the ProtParam tool (http://web.expasy.org/protparam/, accessed on 8 July 2022).

### 4.2. Gene Structure, Chromosomal Mapping and Cis-Acting Element Analysis of Tomato ALs

According to the genome annotation information excavated from the Tomato Genome sequencing project databases, exon–intron structures for *ALs* were displayed using Gene Structure Display Server (GSDS2.0, http://gsds.cbi.pku.edu.cn, accessed on 9 July 2022). Subsequently, the chromosomal locations were visualized using TBtools software (https://github.com/CJ-Chen/TBtools/releases, accessed on 25 July 2023). In addition, the *cis*-elements of the promoter sequences of *ALs* were predicted within the 2 kb upstream regions of tomato *ALs* by PlantCare (http://bioinformatics.psb.ugent.be/webtools/plantcare/html/, accessed on 10 July 2022).

### 4.3. Phylogeny and Conserved Motif Composition

The full-length amino acid sequences of the AL protein sequences from *A. thaliana*, *O. sativa*, and *S. tuberosum* were downloaded from the TAIR and UniProt databases (https://www.uniprot.org/, accessed on 15 July 2022). ClustalX2 was used to generate multiple sequence alignments of all the downloaded AL proteins, and then phylogenetic trees were constructed using the neighbor joining method with 1000 replicates in MEGA11. The phylogenetic tree was visualized with the online software ITOL tree (https://itol.embl.de/, accessed on 16 July 2022). The conserved motifs were determined using the Multiple Em for Motif Elicitation (MEME) online program (http://meme-suite.org/tools/meme, accessed on 16 July 2022) setting the default number of motifs to 20.

### 4.4. Calculation of Ka/Ks Ratoos and Subcellular Localization Analysis

To detect the mode of selection, the ratios of nonsynonymous substitutions per nonsynonymous site (Ka) to the number of synonymous substitutions per synonymous site (Ks) between paralogues were calculated based on coding sequence alignments using DnaSP v5.0 software [40]. CELLO v.2.5 was utilized to investigate the subcellular locations at which the AL proteins were embedded (http://cello.life.nctu.edu.tw/, accessed on 18 July 2022).

### 4.5. Gene Duplication and Synteny Analysis of the AL Gene Family

The gene duplication events of tomato *AL* genes were investigated using MCScanX software (https://github.com/wyp1125/MCScanX, accessed on 25 July 2023) [41]. To analyze synteny with other plants, the total *AL* genes located on different chromosomes were obtained from four tomato species. MCScanX software was applied to explore the microsynteny between *SlAL* genes and *AL* genes of *A. thaliana*, *Z. mays, V. vinifera*, *S. tuberosum*, *S. melongena* and *O. sativa*. Circos software (http://circos.ca/software/, accessed on 25 July 2023) was employed to graphically visualize the syntenic relationships among the four species [42].

### 4.6. Plant Treatments

The tomato cultivar ‘M82’ was used in the present study. Seedlings were grown in a greenhouse at 25 °C (light/dark, 16/8 h). When the seedlings had grown to the three-leaf stage, they were subjected to drought treatment (300 mmol/L mannitol) and salt treatment (200 mmol/L NaCl), and untreated seedlings served as controls. The treated leaves were collected at four time points (0, 1, 6, and 9 h). The samples were stored at −80 °C after being frozen in liquid nitrogen for RNA extraction. Three biological replicates were performed per sample.

### 4.7. qRT-PCR Analysis

A Polysaccharide Polyphenol Plant Total RNA Extraction Kit (Tiangen, Beijing, China) was used to exact the total RNA of collected samples, followed by first-strand cDNA synthesis according to the instructions of 5 × All-In-One RT MasterMix (ABM, Vancouver, Canada). Gene-specific primers were designed using Primer-BLAST (https://www.ncbi.nlm.nih.gov/tools/primer-blast, accessed on 7 July 2022), and *SlActin* (*Solyc03g078400*) was used as the internal control. The detailed PCR primer sequences are shown in Supplementary Table S7. qRT-PCR was performed in a LightCycler96 real-time system (Roche, Basel, Switzerland) using SYBR green master mix (Vazyme, Nanjing, China). Three biological and two technological replicates were used to perform qRT-PCR analysis by using the 2^−ΔΔCT^ method.

### 4.8. Expression Patterns of SlALs

Transcriptome data were used to explain the expression patterns of the *SlAL* family in different tissues (unopened flower buds, flower, leaves, stem, root) and developmental stages of tomato fruit (1 cm, 2 cm, 3 cm, mature green, breaker, and breaker + 10 day fruits) and were downloaded from the Tomato Functional Genomics database (TFGD, http://ted.bti.cornell.edu/, accessed on 25 July 2022) [43]. The heatmap module in TBtools was used to generate heatmaps of *SlALs* expression patterns [44]. The generated heatmap was then beautified using AI software (Adobe Illustrator 2020, https://www.adobe.com/cn/products/illustrator.html, accessed on 11 August 2022).

### 4.9. Subcellular Localization Analysis

The recombinant bacterium GV3101::pSuper1300-SlAL3-GFP was activated, and GV3101::pSuper1300-GFP was injected into tobacco (*Nicotiana benthamiana*) by transient transformation with tomato bush virus P19 protein and HY5 nuclear locus protein in a 3:2:3 volume mixture for 12 h under dark culture, followed by 3 d under normal culture and then by laser confocal microscope. The fluorescence signal was acquired and photographed with ZEN Imaging Software (Version 2.3).

### 4.10. Statistical Analysis

All data are expressed as the mean ± SE. GraphPad Prism 9.0 software was employed for data analysis. One-way ANOVA post hoc Duncan’s multiple range test was used for multiple variable comparisons at a significance level of 0.05.

## 5. Conclusions 

In conclusion, a total of 39 individual members of the *AL* gene family were identified in four species of tomato for the first time. These genes were unevenly distributed on the 12 chromosomes of tomatoes. According to phylogenetic analysis, the *AL* gene family was phylogenetically divided into four clades, bearing parallel conserved motifs and gene structures in the same clade. Gene duplication analysis indicated that the segmental duplication mechanism gave rise to the expansion of the *AL* gene family in tomato and that these genes belong to slowly evolving multigene families. Various *cis*-acting elements participate in abiotic stress, phytohormone responses, and plant growth and development. In addition, RNA-seq and qRT-PCR based expression profiles revealed genes involved in responses to drought and salt stresses and illustrated organ specific expression patterns. Overall, the present study confirmed that *ALs* may play a major role in salt and drought resistance in tomatoes, laying the groundwork for future research on the significance of the *AL* gene family in tomato breeding and resistance enhancement.

## Data Availability

Not applicable.

## Supplementary Materials

The following supporting information can be downloaded at: https://www.mdpi.com/article/10.3390/plants12152829/s1, Figure S1: Phylogenetic tree; Figure S2: Motif result graph; Table S1: *AL* genes candidates identified in tomato; Table S2: Classification and statistics of promoter *cis*-element; Table S3: *Cis*-acting element types, start positions, and termination positions of *ALs* genes. Table S4: Gene duplication, Ks, Ka, and Ka/Ks values calculated for paralogous *AL* gene pairs in tomato; Table S5: Direct homology of *AL* genes in multiple species; Table S6: Expression of *AL* genes in different tissues; Table S7: List of primers used for this article.
