# Supplementary figures and images for: Identification and Expression Analysis of the Alfin-like Gene Family in Tomato and the Role of SlAL3 in Salt and Drought Stresses
Source: Plants (Basel). 2023 Jul 31;12(15):2829. doi: 10.3390/plants12152829 (PMC10421131; doi:10.3390/plants12152829)

**Figure S1.** Phylogenetic tree.

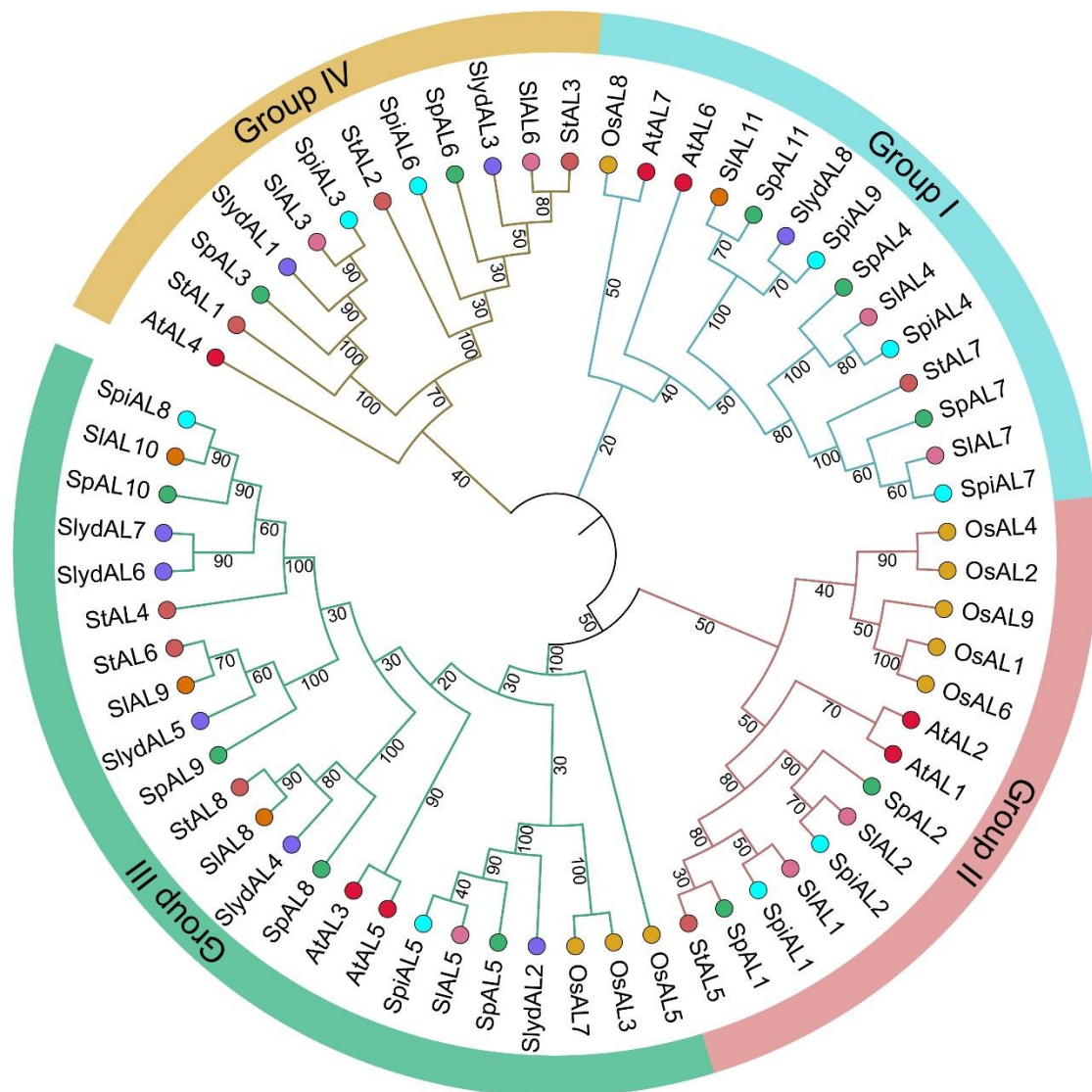

Figure S2. Motif result graph.

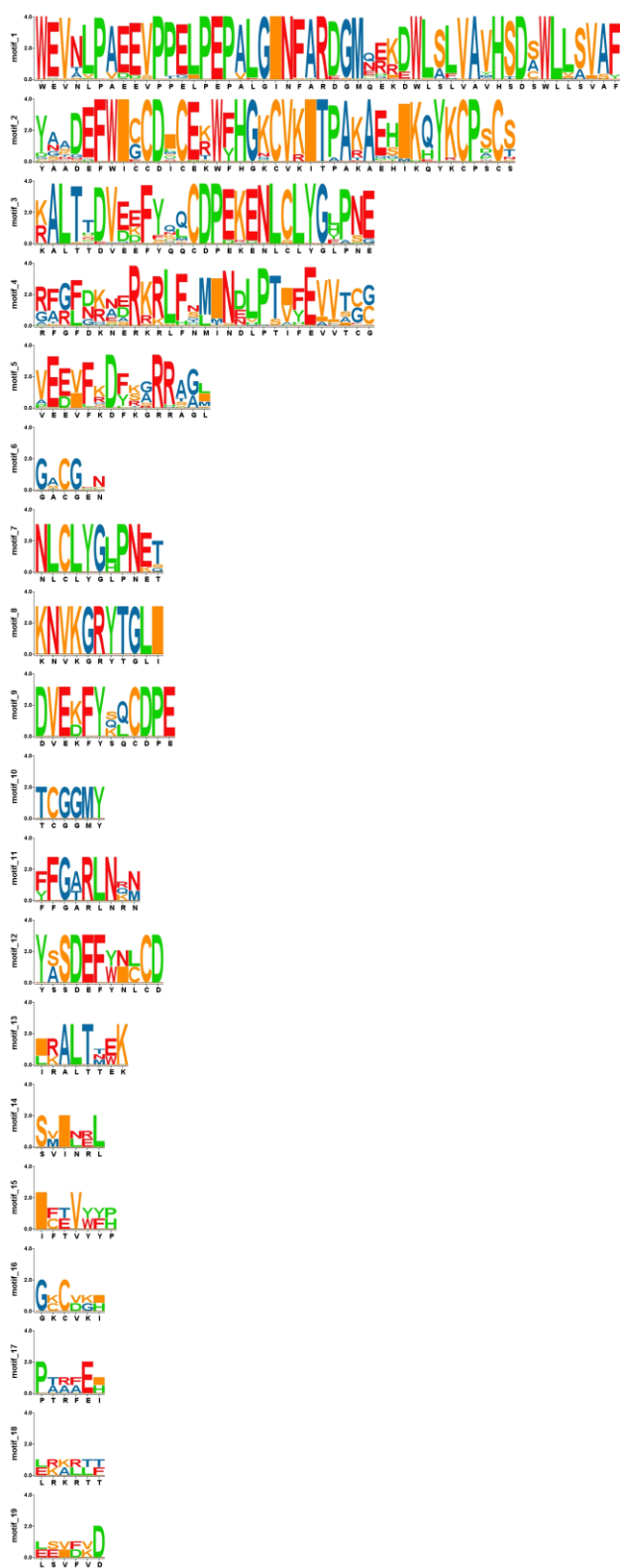

Supplement: Supplementary file 1 [file plants-12-02829-s001.zip › Supplementary figure.pdf]
